# Synthesis of Nano-Paramagnetic Oleuropein to Induce KRAS Over-Expression: A New Mechanism to Inhibit AGS Cancer Cells

**DOI:** 10.3390/medicina55070388

**Published:** 2019-07-19

**Authors:** Farhad Barzegar, Mohammad Zaefizadeh, Reza Yari, Ali Salehzadeh

**Affiliations:** 1 Department of Biology, Rasht Branch, Islamic Azad University, Rasht, Iran; 2 Department of Biology, Ardabil Branch, Islamic Azad University, Ardabil, Iran; 3 Department of Biology, Borujerd Branch, Islamic Azad University, Borujerd, Iran

**Keywords:** AGS Cancer, K-Ras, miR-200 oncogene, nano-oleuropein, oleuropein, real-time PCR

## Abstract

*Background and objectives:* Human gastric adenocarcinoma (AGS) is one of the most common malignant cancers worldwide. The present study aimed to transfer oleuropein into cancer cells using synthetic paramagnetic nanoparticles and study their effect on the AGS (ATCC^®^ CRL1739™) cell line. *Materials and Methods:* Paramagnetic nano-oleuropein was synthesized using four-stage co-precipitation by developing NH-connected bridges and was evaluated by EDS, SEM and FTIR methods. Different concentrations of magnetic oleuropein (0, 0.15, 0.45, 1.37, 4.12, 12.35, 37.04, 111.11, 333.33, 1000 µg/mL) were used to treat the AGS cell line in a completely randomized design using a statistical framework with three replicates. The relative expression rate of miR-200 and KRAS oncogenes was evaluated using real-time PCR. The inhibition rate of the AGS cells was assessed using the MTT test at 24, 48 and 72 h intervals. *Results:* The results showed that there was a significant difference between the inhibition rates of magnetic nano-oleuropein at IC50-24h (23.6 µg/mL), IC50-48h (15.2 µg/mL) and IC50-72h (9.2 µg/mL). Real-time PCR indicated that the relative expression of KRAS and miR-200 genes was highest at IC50 at these intervals. *Conclusions:* Magnetic nano-oleuropein can be subjected to objective testing and clinical evaluations as a natural antioxidant to prevent and treat gastric adenocarcinoma.

## 1. Introduction

Gastric cancer comprises almost 12% of all cancers. It is the fourth most-prevalent cancer and the second cause of cancer mortality worldwide [1]. The highest rates of gastric cancer have been reported in Japan, China and Russia, while developed western countries have the lowest rates [2]. Almost 90% of gastric tumours appear as malignant growths in gastric tissue and can be identified as the intestinal or diffuse type [3]. The intestinal type is the most common [4]. Environmental risk factors that contribute to gastric cancer include *Helicobacter pylori*, lifestyle and nutrition [5]. However, the main components are genetic history and polymorphism [2].

Classification of cancer genes into dominant and recessive tumour suppressor oncogenes has a long history [6]. Oncogenes are caused by mutations in a normal primary gene known as the proto-oncogene. The proto-oncogene can become an oncogene by a relatively small modification in its original function. A mutation within a proto-oncogene, or within a regulatory region, can cause a change in the protein structure, causing an increase in protein activity or loss of regulation. KRAS and MYC are oncogenes related to digestive system cancers, including gastric, pancreatic and intestinal cancers [5]. KRAS (Kirsten rat sarcoma viral oncogene homolog) is a small GTPase that plays an on/off role in guiding intracellular signals [7]. Mutation of KRAS decreases GTPase function [8]. A point mutation in exons 1 and 2 of the KRAS gene is responsible for causing a cell to become malignant [9]. In this regard, 95% of the mutations occur in codons 12 and 13 [10]. Mutation in these codons results in protein changes in RAS leading to increased resistance to GTPase function [11]. Because GTP has more time to connect to the protein, RAS intensifies cell, growth and proliferation of the mutant [11] and activates the RAS/RAF/MAPK and PI3K/AKT pathways [12]. Recently, the KRAS mutation has been reported in several human cancers [13].

It is known that miRNAs are a class of short, small non-coding RNAs that adjust the expression of RNAs. Because miRNA plays a role in differentiation and proliferation, disturbance in the expression of miRNAs might be related to cancer. Several studies confirm that miRNAs play an important role in the onset and progress of cancer. Depending on which mRNAs are inhibited by miRNAs, they can become tumour inhibitors or oncogenes [14].

Little data exists on factors affecting miRNA expression; however, these small adjusting RNAs can be of therapeutic value in the treatment of cancer. In addition, miRNAs are powerful tools in the prognosis, prediction and control of cancer. OncomiRs and tsmiRs (tumor suppressor) are two types of miRNAs in cells. Some oncomiR genes are oncogenes. The dysregulation of certain microRNAs (oncomiRs) has been associated with carcinogenesis, malignant transformation and metastasis [15] Other oncomiR genes are tumour suppressors in a normal cell, so that dysregulation of these genes leads to cancer growth [16,17]. Oleuropein can decrease the expression of some miRNAs such as miR-21 and miR-155 [18].

The extract of the leaves and other parts of olive trees contain polyphenol compounds that have a strong antioxidant function [19]. Oleuropein, with the chemical formula C_25_H_32_O_13_, is the most extractable polyphenol of olive (*Olea europaea*) leaves and fruits. Oleuropein is the primary constituent and the cause of the bitter pungent taste in olives [20].

Oleuropein has pharmacological effects such as antioxidant, anti-inflammation, anti-cancer and antimicrobial activity [21,22]. Oleuropein is an anti-cancer compound that acts through cell obstruction during the G_2_/M phase, leading to tumour recession and inhibition of proliferation [19]. Oleuropein inhibits the proliferation and invasion of A172 and U251 glioma cells by suppression of the AKT signalling pathways. Oleuropein can result in a significant decrease in AKT phosphorylation through the intervention of Bax and inhibition of BCL2 [23]. Oleuropein was used for inhibition of the human hepatoma cell (HCC) line by suppression of the PI3K/AKT pathway [24]. miR-200 can inhibit the AKT and ERK pathways by targeting the 3′UTR region of KRAS gene [25].

Metallic nanoparticles have been used for drug delivery; the attachment of drugs or proteins to metallic nanoparticles. They can produce haemolytic and cytotoxic effects against cancer cell lines [26]. Paramagnetic oleuropein (if synthesized) can be transferred to the desired location via magnetic field conductivity, which is used in anti-cancer materials including cisplatin, iron oxide, and curcumin. Binding of anti-cancer materials to iron nanoparticles was performed using the co-precipitation method via NH_2_-connected bridges. Generally, magnetic nanoparticles have properties that make them ideal for transferring to the targeted positions using a magnetic field [26,27,28,29]. The present study investigated the formation of a ligand between the nanoparticle and oleuropein and likelihood of nano-oleuropein transfer for inhibition of a gastric cancer cell line.

## 2. Materials and Methods

### 2.1. Extracting Olive and Purification of Oleuropein

Olives from the cultivar Mary that were in the conserve stage were collected from the region of Rudbar in Guilan province in Iran. After washing the fruits, their kernels were ground and put into distilled water for rewashing. After 24 h, the water was filtered using 0.2 filters and sterilized. The liquid extract was put into a freezer at −80 °C and freeze-dried. The oleuropein was purified using HPLC-Prep. The resulting powder was freeze-dried and a pure powder was obtained.

### 2.2. Synthesis of Nano-Paramagnetic Oleuropein

#### 2.2.1. Synthesis of Iron Nanoparticles

Wet co-precipitation was used for nano-magnetic synthesis. First, in a 250 mL beaker, 2.15 g of FeCl_2_4H_2_O was mixed with 5.84 g of FeCl_36_H_2_O in 80 mL deionized water to obtain an orange solution. This was stirred for 15 min to completely dissolve the salts. Next, 25 mL of NaOH (3 M) was added dropwise to the solution. A black sediment was observed after adding the first drop and the sediment was completed at a pH of 9. It was stirred for 30 min at 80–90 °C, and then 2.5 g of citric acid was added to 10 mg of water at 90 °C and stirred for 60 min. Next, the sediment was rewashed with deionized water and dried at ambient temperature. The synthesis of nanoparticles takes place as follows (Fe^3+^:Fe^2+^:O^2^ ratio of 2:1:4).

2Fe^3+^ + Fe^2+^ + 8OH^−^ → Fe_3_O_4_ + 4H_2_O

#### 2.2.2. Silication of Outer Surface of Nano-Magnetite

In this stage, 1 g of the magnetic nanoparticle obtained from the previous stage was dissolved in 150 mL of ethanol and then 2 mL of ammonium 0.25%, 20 mL of deionized water and 12 mL of tetraethyl orthosilicate were added. After injecting nitrogen into the solution, it was refluxed at 90 °C for 2 h and precipitated using a magnet after 2 h. The solution was removed and the precipitated solution was washed with distilled water, ethanol, and methanol and then dried at ambient temperature.

#### 2.2.3. Functionalization of Magnetic Nanoparticles Covered with 3-Aminopropyl Trimethoxysilane (3-APTMS)

Magnetic nanoparticles covered with silica were dissolved ultrasonically in 150 mL of water:ethanol (1:1) for 30 min for homogenous distribution. Next, 3 mL of 3-APTMS was added and the reaction began for 2 h at a reflux temperature of 60 °C under N_2_. After 2 h, the magnetic reflux was put under a beaker, and the solution phase was removed. The precipitated solutions were rewashed with distilled water and ethanol and were dried at ambient temperature.

#### 2.2.4. Attachment of Oleuropein to Magnetic Nanoparticles Functionalized with 3-APTMS

Following synthesis and nanoparticle functionalization, the attachment of oleuropein occurred as follows. About 0.35 g oleuropein and 0.15 g dicyclohexylcarboimide (DCC) were ultrasonicated for 1 h in 30 mL DMSO. The resulting mixture was centrifuged and the nanoparticles modified with APTMS were added to the suspension and stirred at room temperature for 12 h. The suspension was centrifuged and the precipitate was washed with distilled water. The co-precipitation method was used to develop magnetite (Fe_3_O_4_) nanoparticles through the precipitation of Fe_2_ and Fe_3_ salts with a stoichiometric ratio of 1:2 in a base solution as: 2FeCl_3_·6H_2_O + FeCl_2_·2H_2_O + 8NaOH → Fe_3_O_4_ + 8NaCl + 8H_2_O. The magnetite properties of the nanoparticles were evaluated using magnets.

### 2.3. Cell Culture

AGS (ATCC^®^ CRL1739™) cell line was supplied by Pasteur Institute of Iran and cultured in RPMI 1640 media with 10% FBS (Gibco, Waltham, MA, USA) and 1% penicillin/streptomycin with a final concentration of 100 units per ml. To provide growth conditions, the cells were incubated at 37 °C, 5% CO_2_ and 95% humidity for 24 h.

### 2.4. MTT Assay

The MTT assay was used to evaluate the proliferation rate of cells. About 8000 cells were cultured in 96 boxed pellets at 37 °C, 5% CO_2_ atmosphere and 95% humidity for 24 h. Next, they were treated with different concentrations (0, 0.15, 0.45, 1.37, 4.12, 12.35, 37.04, 111.11, 333.33 and 1000 µg/mL of nano-magnetitc Oleuropein composite; the content of Oleuropein was 9.8% on the nanocomposite) of Oleuropein at 24 and 48 h and incubated with MTT solution for 4 h. Eventually, the surface fluids were removed, the formazone crystals were dissolved in DMSO and the ELISA plate reader was used to measure the absorbance sample at a wavelength of 570 nm. Magnified images of the cells were examined using an inverted microscope.

### 2.5. RNA Extraction

RNA extraction was done using the TRIzol method. TRIzol and chloroform were added to the treated cells. After incubation and centrifuging, a white protein layer of TRIzol and chloroform developed at the top and bottom of the RNA, respectively. After removing 75% of the bright surface solution and the addition of cold isopropanol solution, the RNA precipitated as a white spot. The quality and quantity of the mRNA were determined using the *NanoDrop* method (Thermo 2000c, Thermo Fisher Scientific, Waltham; MA, USA) at the 260 and 280 nm wavelengths.

### 2.6. Synthesis of cDNA

The cDNA was synthesized using a Qiagen kit and Oligo dT primer for KRAS. Stem-loop primer sequencing was used for miR-200 cDNA synthesis.

### 2.7. Real-Time PCR

SYBR Green (Sinaclon, Tehran, Iran) was used to perform real-time PCR. For this purpose, 10 µL of SYBR Green 1× and 1 µL of cDNA and 0.7 µL of reverse primer were used. The primers are shown in Table 1. Relative analysis at the mRNA level was performed with three replicates using the Pfaffian method with e^−∆∆ct^ compared with the GAPDH housekeeping gene.

### 2.8. Statistical Analysis

MTT and relative gene expression data were used for analysis of variance (ANOVA) and post hoc for comparison of means using the Duncan method test at the 0.01 probability level. Statistical analysis of the data was done with SPSS 24 (v. 24, IBM, Armonk, NY, USA).

## 3. Results

### 3.1. Synthesis and Identification of Oleuropein Attachment to Functionalized Magnetic Nanoparticles

The magnetic nanoparticles covered with silica and functionalized with APTMS were connected to free (C=O, C-OH) oleuropein groups by chemical bonding using the free amino group of APTMS. Moreover, the synthesis of the magnetic nano-oleuropein complex was confirmed by SEM, EDS and FTIR methods.

**Figure 1:**
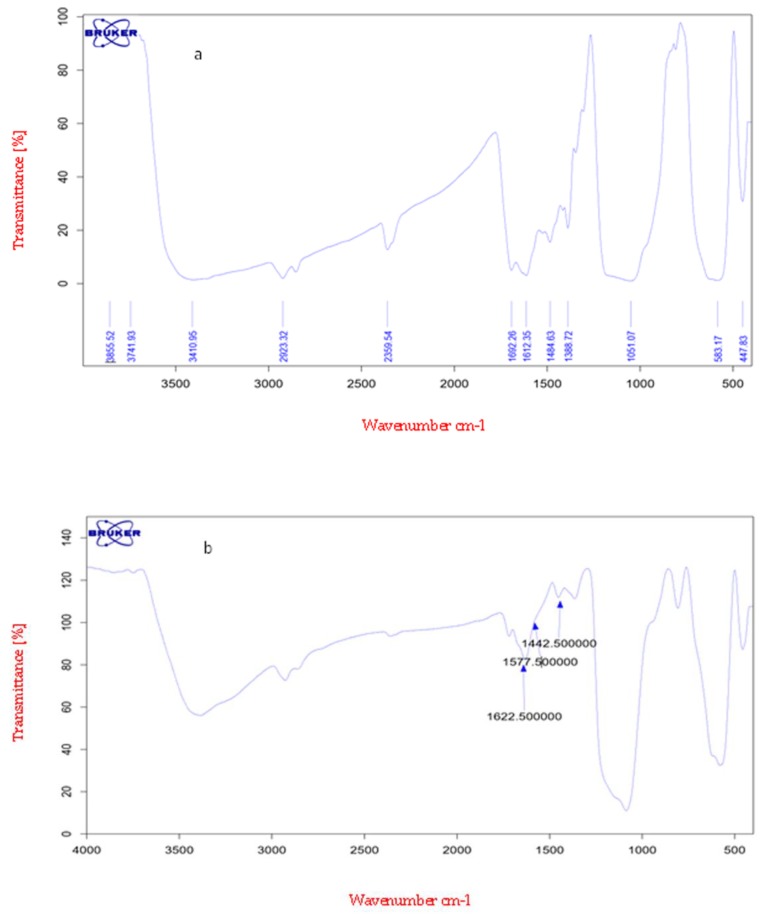
(**a**) FT-IR spectrum of oleuropein. (**b**) Spectrum of magnetic nanoparticles connected to oleuropein (nano-oleuropein).

The FTIR spectra of oleuropein have specific messengers at 1440, 1575, and 1625 cm^−1^. In the FTIR of nanoparticles modified with oleuropein, it was observed that oleuropein successfully coupled with the nanoparticle surface by means of an amide connection between the carboxyl acid groups of the OP and amino groups of APTMS (Figure 1).

**Figure 2:**
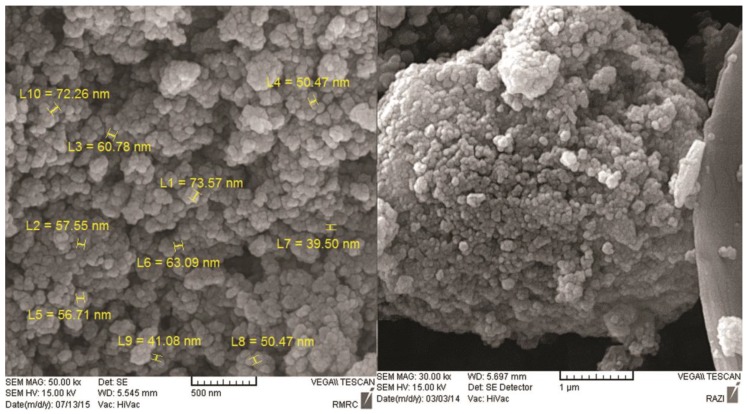
SEM images of magnetite nanoparticles functionalized with 3-amino propyl trimethoxysilane connected to oleuropein at a magnification of 500 nm and 1 µm. About 20% of the particles were <20 nm in size and 70% were 50–100 nm.

**Figure 3:**
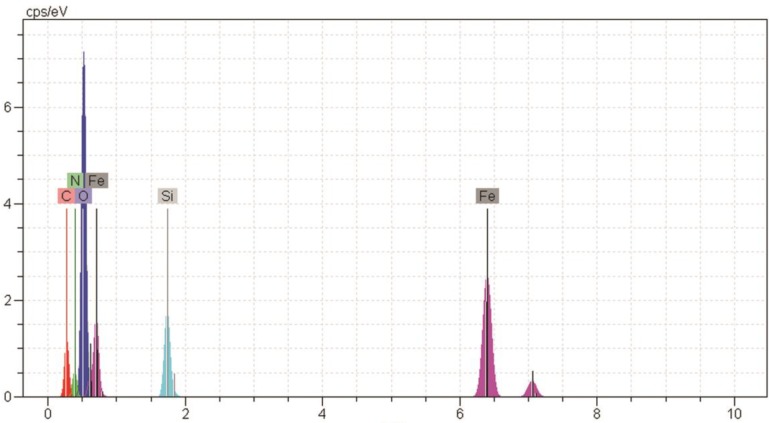
EDS image of magnetite nanoparticles functionalized with 3-amino propyl trimethoxysilane connected to oleuropein.

Figure 2 and Figure 3 show the scanning electron microscope (SEM) and energy dispersive x-ray spectroscopy (EDS) images, respectively, related to magnetite nanoparticles functionalized with 3-amino propyl trimethoxysilane connected to oleuropein. The complex size varied from 39.50 to 73.5 nm. The Fe, Si, N, C, and O ions could be observed in EDS for magnetite nanoparticles functionalized with 3-amino propyl trimethoxysilane connected to oleuropein. The stability of the magnetite oleuropein is presented in Table 2. In addition to the O and Fe elements, C, N and Si were also present in the cells.

### 3.2. Results of MTT Assay

The results of the ANOVA analysis for the survival test performed on the AGS cell line for different concentrations of oleuropein are shown in Table 3 at intervals of 24, 48 and 72 h. There was a significant difference at *p* < 0.01 for different concentrations of oleuropein on growth inhibition of the AGS cell line at all three intervals.

**Figure 4:**
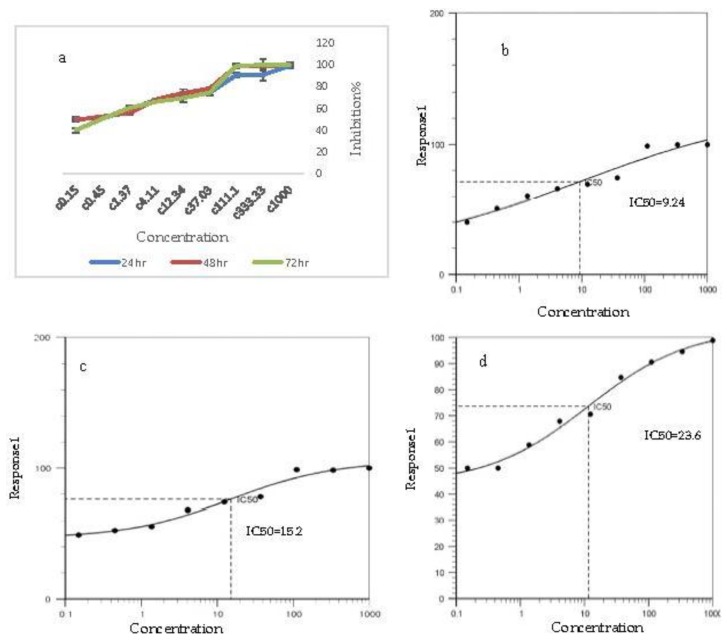
(**a**) Inhibition rate on AGS cancer cells at different concentrations and IC50% concentration of nano-oleuropein. (**b**) IC50% for 72 h after treatment, (**c**) IC50% for 48 h after treatment, (**d**) IC50% for 24 h after treatment.

Comparison of the inhibition rate of nano-oleuropein on AGS cell line growth at the interval of 24 h showed that the inhibition rate was high, even at the lowest dose. Overall, it can be stated that the inhibition rate at IC50 with a concentration of 23.6 µg/mL at 24 h, 15.2 µg/mL at 48 h and 9.2 µg/mL at 72 h reached its highest rate (Figure 4). Triplicate treatment of nano-oleuropein showed that the inhibition of the AGS cell line depends on concentration and that increasing the concentration of nano-oleuropein increases the rate of cell inhibition.

**Figure 5:**
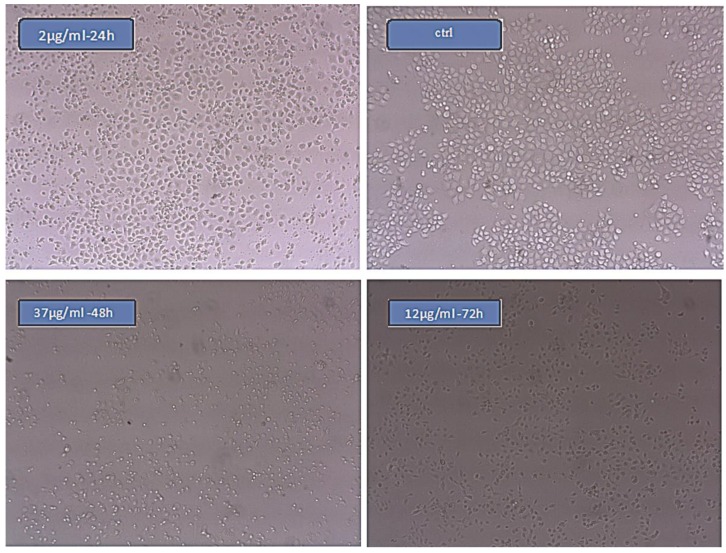
Images of cells after the MTT assay in three concentrations for IC50% nano-oleuropein (24, 15, 10 µg/mL) after 24, 48 and 72 h of treatment.

The images made by inverted microscopy show that the growth inhibition increased along with the concentration of nano-oleuropein (Figure 5). The dead cells in the figure are dark in colour. The ratio of these cells in the sample treated with nano-oleuropein looks significantly higher. These results are in accordance with the results of the MTT assay.

### 3.3. Gene Expression under Nano-Oleuropein Treatment; KRAS and miR-200

The results of ANOVA analysis for KRAS and miR-200 genes indicated that the expression of the genes varied (*p* < 0.01) for different concentrations of nano-oleuropein treatments (Table 4). This means that the change in the expression of KRAS and miR-200 showed a significant change in the concentration of nano-oleuropein.

The Duncan test for comparison of the means of treatment at different nano-oleuropein concentrations in the AGS cell line for KRAS and miR-200 gene expression showed a significant difference (*p* < 0.01) in relative expression of the KRAS gene. Comparison of the mean relative expression of the KRAS gene at different concentrations indicated that the highest expression was observed at 0.15, 0.45 and 1.37 µg/mL and the relative expression of this gene ranked second at 1000, 333.33, 111.11, 37.03, 12.03, and 4.11 µg/mL (Table 5).

The results of the relative expression of miR-200 gene showed a significant difference (*p* < 0.01) between nano-oleuropein concentrations for relative expression of the miR-200 gene. Comparison of the mean relative expression of the miR-200 gene at different concentrations revealed the highest expression at 333.33, 111.11 and 37.03 µg/mL. However, relative expression of this gene was observed at 1.37 µg/mL as well. The minimum relative expression of this gene was seen at 0.15, 0.45, 0.11, and 12.03 µg/mL and the maximum relative expression was seen at 333.33 and 111.11 µg/mL.

Investigation of the effect of concentration for magnetic nano-oleuropein showed that the linear regression between concentration and relative expression of miR-200 was positively significant. Generally, an increase in concentration of nano-oleuropein increased the relative expression of miR-200. The regression equation between the two mentioned variables is as follows:

Y = 0.928 + 0.01x

where Y and x are the relative expression of miR-200 and the concentration, respectively. This relationship was statistically significant (*p* ≤ 0.05).

The linear negative relationship between the relative expression of KRAS and different concentrations of nano-oleuropein was not significant, but the relationship was significant according to the nonlinear compound model. Here, the correlation between the relative expression of the KRAS gene and mir-200 was negative and non-significant (*p* = 0.51; y = −0.27). In other words, although they had an incremental effect on expression of miR-200, different concentrations of nano-oleuropein had a cut-off point for expression of KRAS.

## 4. Discussion

The aim of synthesizing this complex was to target the tumor site with oleuropein using a magnetic system. The magnetite nano-oleuropein was synthesized with a size of 39.50 to 73.57 nm by co-precipitation and the NH-connected bridges method. The synthesized magnetic nano-oleuropein was confirmed by EDS, SEM and FTIR spectroscopy. Laurent et al. (2014) [27] used a nano-oxide of magnetic iron to inhibit cancer cells by directed transfer of drugs under in vivo and in vitro conditions. Previous studies have synthesized the nanoparticles of cisplatin [28], iron oxide [27], curcumin [29], anthocyanine and vitamin C. The results of these studies were consistent with the present study.

Magnetic nano-oleuropein led to the inhibition of AGS cells after 24, 48, and 72 h of post-treatment. The inhibition rate was higher at 48 and 72 h than 24 h. The inhibition was also significant at 24 h (*p* < 0.01) and showed that the inhibition was the apoptosis type.

The mechanism of the inhibition of cancer cells was evaluated by using the hypothesis of interpretation of KRAS oncogene expression. Investigation of the relative expression of KRAS also showed higher expression in the AGS cancer cell line. At the 0.15 µg/mL concentration, the expression was 21.69 times greater than that for AGS cells before treatment. Despite the inhibition of cells by oleuropein treatment, increased expression of KRAS with nano-oleuropein was not expected. It was expected that the cells would show more proliferation, but they were primarily inhibited. This observation may explain the regulatory system of gene expression after transcription, because increased KRAS and its simultaneous inhibition of KRAS mRNA occur by miR-200 expression. The increasing miR-200 expression caused KRAS mRNA degradation and decreased mRNA for KRAS, which led to the inhibition of cell proliferation [25]. In other words, the inhibitory structure of KRAS mRNA caused the production of double-stranded mRNA through the increased number of transcriptions of miR-200 and its hybridization with the 3′UTR region, leading to its degradation [25,30].

The second probable mechanism of controlling cancer cells by magnetic nano-oleuropein is through the increased relative expression of KRAS. An increase in KRAS expression and production of the related protein may lead to increasing CIN in cancer cells [31], which may destroy the DNA at the chromosome surface, or part of it, leading to cell apoptosis and inhibition. Although this hypothesis does not correspond with miR-200 expression, it should be investigated. Regarding the inhibition of AGS cancer cells by magnetic nano-oleuropein and proof of its action and mechanism in vivo, it can be used as a targeted transfer using a magnetic field [27,28].

## 5. Conclusions

Magnetic nano-oleuropein was successfully synthesized and characterized. The size of the product was >70 nm. It was found that this magnetic nano-oleuropein could trigger apoptosis in the AGS cell line. The magnetic nano-oleuropein increased KRAS expression and inhibited the miR-200 gene.

## Figures and Tables

**Table 1:** The sequence of primers for KRAS and miR-200 genes.

Name of Genes	Sequence of Primers
*KRAS*	5′-GTGGTAGTTGGAGCTTGTGG-3′ 5′-TGACCTGCTGTGTCGAGAAT-3′
*miR-200*	5′-GTTTGTGACGACCCATTCTGC-3′ 5′-GAGCCTGGGACGTGACC-3′
*GAPDH*	5′-AGG GCT GCT TTT AAC TCT GGT-3′ 5′-CCC CAC TTG ATT TTG GAG GGA-3′

**Table 2:** Outcome of elemental data analysis of Figure 3.

Elements	Unn. C Wt.%	Norm C Wt.%	Atom C At%
Carbon	5.53	5.28	11.55
Nitrogen	5.52	5.28	9.89
Oxygen	30.65	29.28	48.04
Silicon	5.05	4.82	4.51
Iron	57.94	55.34	26.02

**Table 3:** Outcome of ANOVA analysis for the MTT test (*p* < 0.01).

S.O.V	DF	Means of Square (MS)		
		After 24 h	After 48 h	After 72 h
Concentration	8	0.133 **	0.515 **	0.351 **
Error	18	0.03	0.05	0.159

** Significant difference at the 0.01 level of probability.

**Table 4:** Outcome of ANOVA analysis for KRAS and miR-200 genes (*p* < 0.01).

S.O.V	DF	MS (Means Square) *miR-200*	MS (Means Square) *KRAS*
Concentration of Nano-Oleuropein (T)	8	5.243 **	32.81 **
Error (E)	18	0.496	10.08

** significant difference at the 0.01 level of probability.

**Table 5:** Comparison of the means of treatment at different concentrations of nano-oleuropein on AGS cells relative to KRAS and miR-200 genes.

Concentration of Nano-Oleuropein (µg/mL)	Means Relative miR-200 Expression	Means Relative KRAS Expression
1000	3.51 ^a^	0.41 ^c^
333.33	3.48 ^a^	0.43 ^c^
111.11	4.78 ^a^	0.73 ^c^
37.03	1.09 ^b^	0.42 ^c^
12.03	0.63 ^b^	1.95 ^b^
4.11	0.15 ^b^	0.14 ^c^
1.37	1.19 ^b^	20.8 ^a^
0.45	0.68 ^b^	20.6 ^a^
0.15	0.53 ^b^	21.6 ^a^

Non similar alfabetic (a, b, c) significant difference (*p* < 0.01) in the Duncan test.
